# PReS-FINAL-2236: Continuous autoinflammatory syndromes: a single-center experience in Argentina

**DOI:** 10.1186/1546-0096-11-S2-P226

**Published:** 2013-12-05

**Authors:** RA Russo, MM Katsicas

**Affiliations:** 1 Immunology & Rheumatology, Hospital De Pediatría Garrahan, Buenos Aires, Argentina

## Introduction

Patients with autoinflammatory syndromes may present a clinical course characterized by recurrent, episodic manifestations (such as fever, skin rash or visceral involvement) or they may show a continuous, unremitting disease course with persistent clinical manifestations. Patients with certain diseases, such as CAPS or Blau syndrome, usually present this type of course.

## Objectives

To describe the clinical and genetic features of patients with continuous-course autoinflammatory syndromes followed in a tertiary, pediatric hospital.

## Methods

Ad-hoc data bases from our autoinflammatory syndromes clinic were reviewed. Patients attended this clinic between May 2009 and May 2013. Demographic, clinical, laboratory and genetic data were retrieved. Autoinflammatory syndrome was defined as the presence of a chronic, systemic disease with no evidence of malignancy, infection or autoimmunity. Patients with a continuous disease course (persistent clinical manifestations with no free interval and with possible recurrent exacerbations prior to the initiation of therapy) were included in the analysis. Patients with a diagnosis of systemic juvenile arthritis were excluded. Genetic analysis was performed in different locations.

## Results

Fourteen children (9 boys) with a continuous disease course were identified among patients with autoinflammatory syndromes. Median age at presentation: 6 months; median age at diagnosis: 39 months; median follow-up time: 5 years. Two patients had a positive family history. Systems involved: constitutional (fever/weight loss/malaise) 14 patients, skin 14, joints 13, CNS 8, gastrointestinal 7, eyes 7, bone 6, mucosae 5, respiratory 4, muscle 2. Acute phase reactants remained permanently elevated in 10 patients. Growth was impaired In 10 children. Eleven patients showed some degree of disability. Clinical diagnosis were: Blau syndrome (5 patients), CAPS (4), MKD (1), DIRA (1), CRMO (1), CANDLE (1), TRAPS (1). Genetic analysis was performed in 12 patients: pathogenic mutations were found in 7 (4 Blau, 2 CAPS, 1 MKD). Patients were treated with different agents that included steroids (11 patients), methotrexate (9), anti TNF agents (5), and anti IL-1 agents (5). Eleven patients improved (8 of them on biologics), 2 patients remained stable and 1 patient died (suspected DIRA).

## Conclusion

Continuous autoinflammatory síndromes are severe systemic diseases that affect growth and functional capacity of patients. Genetic diagnosis may provide definite diagnosis in a proportion of patients. Therapy with biological agents leads to better outcomes.

## Disclosure of interest

None declared.

